# Conjugation and transposon mutagenesis of *Xenorhabdus griffiniae* HGB2511, the bacterial symbiont of the nematode *Steinernema hermaphroditum* (India)

**DOI:** 10.17912/micropub.biology.000772

**Published:** 2023-04-25

**Authors:** Omar S. Alani, Mengyi Cao, Heidi Goodrich-Blair, Jennifer K. Heppert

**Affiliations:** 1 Department of Microbiology, University of Tennessee at Knoxville, Knoxville, Tennessee, United States; 2 California Institute of Technology, Pasadena, California, United States

## Abstract

Symbiosis, the beneficial interactions between two organisms, is a ubiquitous feature of all life on Earth, including associations between animals and bacteria. However, the specific molecular and cellular mechanisms which underlie the diverse partnerships formed between animals and bacteria are still being explored. Entomopathogenic nematodes transport bacteria between insect hosts, together they kill the insect, and the bacteria consume the insect and serve as food source for the nematodes. These nematodes, including those in the *Steinernema* genus, are effective laboratory models for studying the molecular mechanisms of symbiosis because of the natural partnership they form with *Xenorhabdus* bacteria and their straightforward husbandry. *Steinernema hermaphroditum* nematodes and their *Xenorhabdus griffiniae* symbiotic bacteria are being developed as a genetic model pair for studying symbiosis. Our goal in this project was to begin to identify bacterial genes that may be important for symbiotic interactions with the nematode host. Towards this end, we adapted and optimized a protocol for delivery and insertion of a *lacZ-* promoter-probe transposon for use in the *S. hermaphroditum* symbiont, *X. griffiniae* HGB2511 (Cao et al., 2022). We assessed the frequencies at which we obtained exconjugants, metabolic auxotrophic mutants, and active promoter- *lacZ* fusions. Our data indicate that the Tn *10* transposon inserted relatively randomly based on the finding that 4.7% of the mutants exhibited an auxotrophic phenotype. Promoter-fusions with the transposon-encoded *lacZ* , which resulted in expression of β-galactosidase activity, occurred in 47% of the strains. To our knowledge, this is the first mutagenesis protocol generated for this bacterial species, and will facilitate the implementation of large scale screens for symbiosis and other phenotypes of interest in *X. griffiniae* .

**Figure 1. Creation of Xenorhabdus griffinae transposon mutants and screening for promoter- lacZ expression and auxotrophies:**
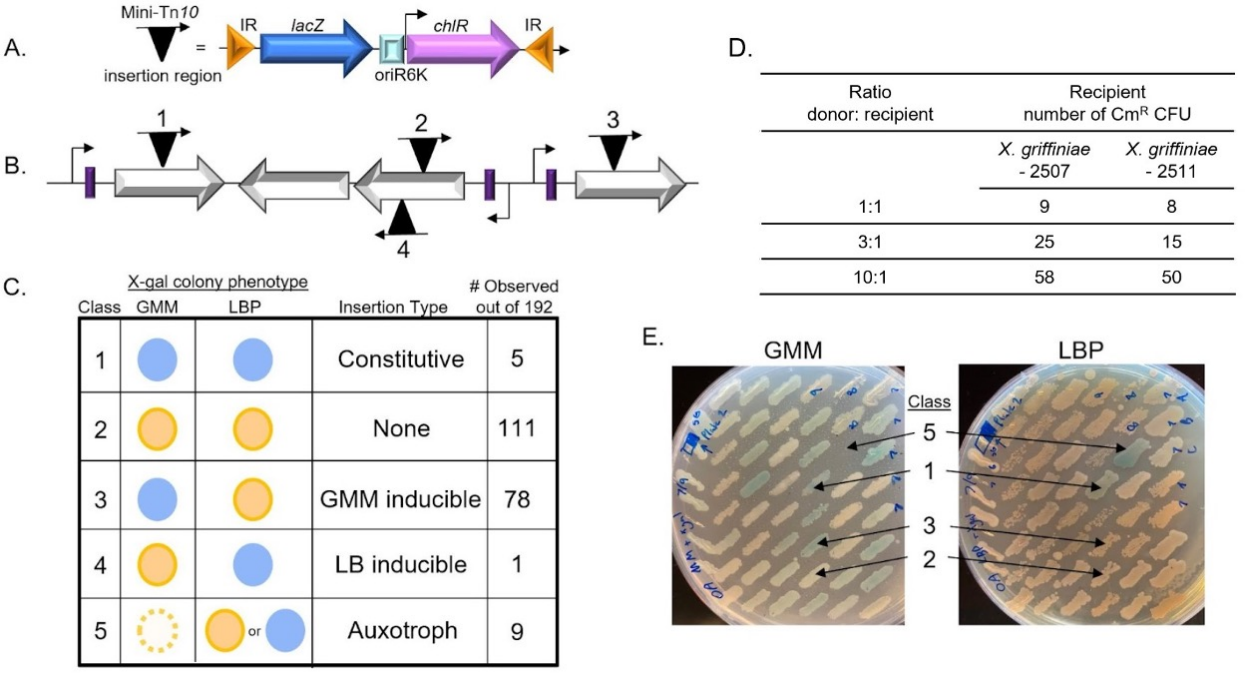
**A)** A region of the pKV124 plasmid carrying a mini-Tn *10* transposon with a promoter-less *lacZ* gene, the *pir* -dependent *ori* R6K and a chloramphenicol resistance cassette flanked by inverted repeats (IR) consisting of the outermost 70-bp of IS *10* R. **B)** Examples of four types of possible mini-Tn *10* transposon insertions (black triangle with orientation of *lacZ* denoted by arrow), relative to coding regions (block arrows) and promoters (purple boxes with arrows indicating direction of transcription) of endogenous genes. Insertions 1, 3, and 4 may give rise to *lacZ* expression driven by the upstream promoters. Insertion 2 is not expected to yield *lacZ* expression, as it is not downstream of a promoter in the genome. **C)** Schematic representation of the types of *lacZ* expression and growth phenotypes that can be observed among mini-Tn *10-lacZ* mutants. Whether *lacZ* is expressed or not is indicated by blue or tan colony color, respectively, when grown on media (e.g., glucose minimal media [GMM] or LB pyruvate [LBP] containing the X-gal substrate for β-galactosidase. Colony color comparisons among media types can indicate whether a promoter is constitutive (expressed on all media tested), inducible (expressed only on certain media) or not expressed. Growth of a mutant on LBP but not GMM suggests the transposon insertion has disrupted a locus required for the biosynthesis of essential nutrients present in LBP, but not GMM. Shown are the numbers of mutants among the 192 *X. griffiniae* mutants tested that exhibited each of the phenotypes observed in this study. **D)** Conjugation conditions were optimized by testing different ratios of two *X. griffiniae* HGB2507 and HGB2511 with the *E. coli* donor strain. Based on the observation that the highest numbers of exconjugants were obtained at a ratio of 1:10 ( *X. griffiniae* : *E. coli* ), this ratio was used for subsequent isolation of *X. griffiniae* HGB2511. **E)** Representative GMM and LBP plates on which individual mutants were patched in parallel to compare phenotypes. The mutants patched included those with phenotype classes of constitutive *lacZ* expression (class 1), no *lacZ* expression (class 2), GMM inducible *lacZ* expression (class 3), LB inducible *lacZ* expression (class 4), and auxotrophy (class 5).

## Description

Entomopathogenic nematodes are roundworms that, together with their bacterial symbionts, parasitize and kill insects, utilizing the bacteria as a food source (Mucci et al., 2022) . These nematodes are both economically and scientifically important since they can be used as biocontrol agents against different insect pests and are used as a model to better understand the molecular basis of animal-microbe symbioses (Koppenhöfer et al., 2020; Eleftherianos and Heryanto, 2020; Heppert et al., 2022). Over the past two decades, bacterial genes and pathways involved in both insect virulence and nematode mutualism have been identified, with the majority of efforts focusing on a few *Xenorhabdus-Steinernema* species pairs, particularly *Xenorhabdus nematophila* and *Steinernema carpocapsae* (Herbert and Goodrich-Blair, 2007; Hasan et al., 2019; Stock, 2019; Cao and Goodrich-Blair, 2020; Lefoulon et al., 2022) . However despite many efforts, reverse genetics and transgenesis in the nematode *Steinernema carpocapsae* have not succeeded. A recently isolated strain of *Steinernema hermaphroditum* is an attractive candidate for nematode genetic engineering because, unlike most Steinernematidae including *S. carpocapsae* which are dioecious, *S. hermaphroditum* is hermaphroditic with rare males (Bhat et al., 2019) . For this reason, *S. hermaphroditum* and their symbiotic bacterial partner *Xenorhabdus griffiniae* are being explored as a new genetic model system for studying symbiosis (Cao et al., 2022) . Towards this goal, we sought to adapt genetic tools to investigate the biology of *X. griffiniae* . Since *Xenorhabdus* strain variability can influence host interactions (Murfin et al., 2015a, Murfin et al., 2015b, Ciezki et al., 2015; Mollah et al., 2020) and genetic tractability (Cuív et al., 2015), we focused on two strains of *X. griffiniae* , HGB2507 ( *S.h.* isolate 5) and HGB2511 ( *S.h.* isolate 9), isolated concurrently from the same population of the *S. hermaphroditum* nematode partner (Cao et al., 2022) . Here we describe optimization of conjugation and transposon mutagenesis in these *X. griffiniae* strains.

To determine if *X. griffiniae* strains are amenable to conjugation and transposon mutagenesis, we used a conjugatable oriR6K suicide plasmid, pKV124, carrying a mini-Tn *10* transposon and a promoter-less *lacZ* gene (Visick and Skoufos, 2001) ( Fig. 1A ). In the standardized conjugation protocol for *X. nematophila,* ampicillin (or carbenicillin can be used interchangeably) is utilized for selecting *Xenorhabdus* bacteria over *E. coli* donor cells *,* because *X. nematophila* is naturally resistant to ampicillin (Gotz et al., 1981) . To apply this protocol, we first tested the carbenicillin resistance among ten isolates of *X. griffiniae* (Table 1). Our data show that *X. griffiniae* exhibited variability in their resistance against carbenicillin among different isolates, suggesting strain-to-strain variation (or phenotypic variation) could occur among these isolates. To facilitate the selection of *X. griffiniae* from *E. coli* donor cells without the use of negative antibiotic selection, we adapted the existing conjugation protocols available for *X. nematophila* to deliver this plasmid from diaminopimelic acid (DAP)-dependent *E. coli pir* -encoding donor cells into *X. griffiniae* recipient cells. After donor and recipient mating, *X. griffiniae* exconjugants were selected by virtue of their resistance to chloramphenicol and their ability to grow in the absence of DAP ( Fig. 1B ). Because at least some *Xenorhabdus* can efficiently kill *E. coli* (Muangpat et al., 2020; Hillman, 2020), we varied the ratio of *E. coli* donor to *Xenorhabdus* recipient in our conjugations. We tested three different recipient to donor ratios; 1:1, 1:3, and 1:10 and found that a 10-fold higher concentration of *E. coli* donor relative to *X. griffiniae* recipient, yielded the highest numbers of exconjugants ( Fig. 1D ). Exconjugants from a total of five plates from a single conjugation were picked and streaked multiple times to ensure clonal purity (see Methods). For this pilot optimization effort, a total of 192 individual exconjugants were picked from selection plates and arrayed in 2 x 96 well plates with LB medium and chloramphenicol, grown overnight at 30°C, and frozen at -80˚C with 20% glycerol for ease of phenotypic screening and subsequent recovery of mutants of interest.

The success of transposition was assessed by estimating frequencies of two phenotypes associated with genome disruption by the Tn *10* transposon: auxotrophy caused by disruption of biosynthetic loci and expression of *lacZ* caused by insertion downstream of active promoters. Each exconjugant was retrieved from the 96-well plates and streaked in a grid format onto either LB pyruvate (LBP) or Glucose Minimal Medium (GMM) with 5-Bromo-4-chloro-3-indolyl β-D-galactopyranoside (X-gal), a chromogenic substrate for β-galactosidase. Auxotrophs were identified as those that grew on LBP but not on GMM. Note that some carryover of nutrients can occur when streaking from LBP to GMM, which may allow some growth of auxotrophs. Mutants with *lacZ* insertions downstream of an active promoter were identified as those with blue color on either substrate ( Fig. 1C ).

All 192 exconjugants tested grew on LBP, but 9 (4.7%) failed to grow on GMM. This is consistent with frequencies of auxotrophic mutants observed in other bacteria such as *Bacillus subtilis* (Youngman et al., 1983) , although higher than the 1-2% observed for *Salmonella enterica* and *Escherichia coli* ( Fig. 1C ) (Kleckner et al., 1975; Nichols et al., 2011) . Based on these previous reports, we would expect ~2% (approximately 4/192) of insertions within genes to cause an auxotrophy. The higher than expected frequency of auxotrophs among the *X. griffiniae* HGB2511 exconjugants tested here may be due to the presence of sibling clonal colonies, derived from replication of mutants prior to plating. Sibling clonal colonies can be reduced by decreasing the amount of time that cells are permitted to grow after conjugation and before selection, and by using independent conjugations to build mutant libraries. It is also possible that if we allowed the exconjugants to grow longer on the GMM, some of the 9 mutants we identified would eventually grow.

During growth on X-gal, 12/192 (6.2%) and 83/192 (43%) exconjugants produced blue color indicative of β-galactosidase activity on LBP and GMM, respectively ( Fig. 1C ). This indicates that the Tn *10* can insert in an orientation that places the *lacZ* gene downstream of active promoters. The successful identification of exconjugants that displayed GMM-induced β-galactosidase activity (relative to LBP) demonstrates the feasibility of screening an *X. griffiniae* library for conditionally-active promoters using pKV124-mediated transposition. Additionally, the locations of two of the Tn *10* insertions were mapped using arbitrary PCR with pKV124-miniTn *10* transposon-specific primers (see Methods, Mapping). When comparing the Tn *10* flanking sequences to the *Xenorhabdus* BMMCB genome available on the Magnifying Genomes platform (MaGe, Vallenet et al., 2013) we found that one of the Tn *10* insertions was within the coding region of a gene with sequence similarity to type III restriction enzymes (LDNM01_v1_400040). The other insertion was in an intergenic region upstream of a gene with homology to a glycosyltransferase family 4 protein within a predicted LPS biosynthesis locus (LDNM01_v1_1170034). Each of the Tn *10* insertion candidates tested returned an unambiguous sequencing result, suggesting that they may be single insertions. However, this is not definitive, and there is still the possibility that we have multiple transposon insertions within the genomes of the library mutants we isolated. Future follow up experiments, such as Southern blotting or whole genome sequencing of representative mutants, will be necessary to ensure that in general we have one transposon insertion per isolate. These results establish an effective conjugation and mutagenesis protocol for *X. griffiniae* symbionts of *S. hermaphroditum* nematodes *.*

## Methods

*Strains and plasmids*

*X. griffiniae* recipient strains were isolated from *S. hermaphroditum* (India) (Bhat et al., 2019) as described in Cao et al., 2022. Two isolates (HGB2507 and HGB2511) were tested for conjugation and transposition. The *E. coli* donor strain (HGB1333) used for this study is pKV124-miniTn *10* transformed into BW29427 (also known as WM3064) (Visick and Skoufos, 2001, and Dehio and Meyer, 1997) . BW29427 requires diaminopimelic acid (DAP) to grow in LB (Dehio and Meyer, 1997) and carries the *pir* gene, encoding the protein *π* necessary for replication of *oriR6K* plasmids, such as pKV124-miniTn *10* (Visick and Skoufos, 2001) . The DAP requirement facilitates counter-selection against the *E. coli* donor by removal of DAP from the medium.

*Conjugation*

Conjugation conditions were determined and optimized for 2 different isolates of *X. griffiniae* with *E. coli* carrying the pKV124-miniTn *10* . A more detailed protocol is provided below. For optimization, each of the two *X. griffiniae* isolates were mixed with the *E. coli* donor strain at different ratios of *X. griffiniae* to *E. coli* (1:1, 1:3, 1:10). The mixtures were then spotted on to LBP + DAP plates and grown for 3 days, after which they were plated on LBP + 15µg/ml chloramphenicol for selection. The colonies were confirmed to be *X. griffiniae* through their distinctive rust color compared to cream-colored *E. coli.* Individual exconjugants (192) of *X. griffiniae* HGB2511 were streaked for isolation on LB with 15µg/ml chloramphenicol and inoculated into a 96 well plate with dark LB and 15µg/ml chloramphenicol, and frozen at -80˚C.

*Phenotype screening*

LBP solid media were made by dissolving yeast extract (5g), tryptone (10g), NaCl (5g), pyruvate (1g), and agar (20g), in 1 Liter of ddH _2_ O and autoclaving prior to pouring plates (~25ml each). GMM solid media were made by combining a salts solution consisting of: nicotinic acid (100mg), KH _2_ PO _4_ (3g), K _2_ HPO _4_ (7g), (NH _4_ ) _2_ SO _4_ (2g), pH to 7.0 using NaOH and H _2_ O added to 500ml; and an agar solution consisting of: 15g Agar, 1g sodium pyruvate, and 450ml H _2_ O. After autoclaving (30min) and cooling until just warm to the touch, the salts and agar solution were combined a with 10ml of a 0.2µm filter-sterilized solution of SL4 salts, 9.1g glucose, 0.05g MgCl _2_ ᐧ(H _2_ O) _6_ , (this solution was mixed, heated to dissolve, and filter-sterilized prior to addition to the salt and agar solution mixture). The GMM plates were then poured at a volume of ~25ml per plate. Plates were top-treated with 50μl of a 10mg/ml X-gal stock solution, resulting in a final concentration of 40μg/ml. Bacteria were streaked onto plates in a grid pattern and were incubated for 2 days at 30˚C and then examined. By visualizing the plates, pigments that expressed any shade of blue, dark or light blue, were labeled as blue clones expressing *lacZ.* For the pigments with no blue shading, which included ones that were yellow, brown, and cream colored, they were labeled as non- *lacZ* expressing clones. Any streak that showed any growth was counted as a grown clone, while those who expressed no growth (not a single colony) were counted as clones that did not grow ( Fig. 1E ).

*Transposon Insertion Mapping*

For two exconjugate clones, the location of mini-Tn *10* transposon insertions in the *X. griffiniae* genome were mapped using arbitrarily-primed polymerase chain reaction (PCR) following the protocol from Saavedra et al., 2017. Briefly, the exconjugates were inoculated from glycerol stocks into 5ml dark LB + 15μg/ml chloramphenicol and grown at 30˚C overnight. Genomic DNA was extracted using the PureLink™ Genomic DNA Mini Kit (Invitrogen) per the manufacturer instructions. In the nested PCR reactions, 40ng of genomic DNA was used in the first round of PCR with primers pkv124_F_1: 5’-GCATCTGCCAGTTTGAGG; ARB6: 5’-GGCCACGCGTCGACTAGTACNNNNNNNNNNACGCC, an annealing temperature of 30˚C and extension time of 1 minute; and 20ng of DNA was used in the second round of PCR with primers (pkv124_F_2: 5’-GCGATTAAGTTGGGTAACGCCAG ; ARB2: 5’-GGCCACGCGTCGACTAGTAC) an annealing temperature of 60˚C and extension time of 1:20 minute. Ten microliters of the products from both rounds of PCR reaction were visualized on a 1.5% agarose gel and then the remaining product (40μl) was purified using the DNA Clean & Concentrator kit (Zymo Research). The products of the second reaction were then sequenced using Sanger Sequencing, and *X. griffiniae* genomic DNA sequences were identified by sequence similarity search using the BLASTN algorithm (Altschul et al., 1990) against the *Xenorhabdus* BMMCB genome LDNM01.1 on the MAGE Microscope platform (Vallenet et al., 2013) .

**Conjugation Protocol:**

**Step 1.** Streak donor and recipient strains onto plates from glycerol stocks stored at -80˚C and grow for 2 days at room temperature or 1 day at 30˚C. This step is necessary because strains, especially *Xenorhabdus* , do not always grow well in liquid media when inoculated straight from freezer stocks.

- *Xenorhabdus* should be grown on media kept in the dark (e.g., dark LB) or supplemented with 0.1% pyruvate (e.g., LBP) because *Xenorhabdus* do not grow well in light-exposed media due to the buildup of toxic oxygen species (Xu and Hurlbert, 1990) .
- Add DAP at 0.3mM final concentration (57μg/ml) to LB for growth of DAP-requiring donor *E. coli* strains. Antibiotics should be added to *E. coli* donor strains to select for the plasmid to be conjugated. To maintain pKV124-miniTn *10,* use 30μg/ml chloramphenicol.

**Step 2.** Inoculate a heavy streak of bacteria (through a dense streak or multiple colonies) from the plates into 5ml dark LB in 2 tubes, one containing *E. coli* and one containing *Xenorhabdus* . Supplement *E. coli* medium with 30μg/ml chloramphenicol, 0.3mM DAP, and 150μg/ml streptomycin. Grow *Xenorhabdus* overnight at 30˚C and *E. coli* overnight at 37˚C.

**Step 3.** Subculture each strain separately into fresh dark LB media without antibiotics. Subculturing is typically done at 1:10 (500μl in 5ml), but can be done at a different dilution, e.g., 1:5 (1000μl in 5ml), to ensure timely growth of strains for use in Step 4. Supplement *E. coli* medium with 0.3mM DAP. Monitor growth using a spectrophotometer at Abs _600_ starting around 2 hours post inoculation.

**Step 4.** When an optical density (OD _600_ ) of ~0.8 is reached, mix donor and recipient together in an eppendorf tube at the desired ratio (e.g., 1:1, 1:3, 1:10) to a total volume of 1200μl. Spin the cell suspension in a benchtop microfuge at maximum speed at room temperature in order to pellet the cells. Resuspend in 25μl dark LB and spot the entire volume onto an LBP plate with 0.3mM DAP. (Do not add antibiotics at this step.) Incubate plates for 2 days at room temperature or overnight at 30˚C (If no colonies grow, allow plates to grow an additional day at 30˚C).

**Step 5.** Using a sterile stick or loop, scrape up the bacterial growth and resuspend well in 1ml dark LB in an eppendorf tube and mix by pipetting and vortexing until no clumps of bacteria remain. Dilute 1:10 by taking 100 μl of cells and transferring to an eppendorf tube with 900μl dark LB. Plate 100μl of both the original resuspension and the 1:10 dilution onto separate on LBP supplemented with antibiotic, in this case 15μg/ml chloramphenicol to select for *Xenorhabdus* with miniTn *10* transposon insertions, and spread across the surface of the plate using a sterile spreader or sterile glass beads. Leave out the DAP at this stage to select against growth of the *E. coli* donor.

**Step 6.** Incubate plates for 2 days at room temperature or overnight at 30˚C, and then select individual colonies for further validation.

## Extended Data

Description: Table 1: Carbenicillin resistance test among X. griffiniae isolates. Resource Type: Image. DOI: 10.22002/whdjy-csp86
